# Device-Free Localization via an Extreme Learning Machine with Parameterized Geometrical Feature Extraction

**DOI:** 10.3390/s17040879

**Published:** 2017-04-17

**Authors:** Jie Zhang, Wendong Xiao, Sen Zhang, Shoudong Huang

**Affiliations:** 1 School of Automation & Electrical Engineering, University of Science and Technology Beijing, Beijing 100083, China; zhangjie2009622@163.com (J.Z.), wdxiao@ustb.edu.cn (W.X.); 2 Centre for Autonomous Systems, Faculty of Engineering and Information Technology, University of Technology Sydney, Ultimo, NSW 2007, Australia; shoudong.huang@uts.edu.au

**Keywords:** device-free localization, received signal strength, extreme learning machine, parameterized geometrical feature extraction

## Abstract

Device-free localization (DFL) is becoming one of the new technologies in wireless localization field, due to its advantage that the target to be localized does not need to be attached to any electronic device. In the radio-frequency (RF) DFL system, radio transmitters (RTs) and radio receivers (RXs) are used to sense the target collaboratively, and the location of the target can be estimated by fusing the changes of the received signal strength (RSS) measurements associated with the wireless links. In this paper, we will propose an extreme learning machine (ELM) approach for DFL, to improve the efficiency and the accuracy of the localization algorithm. Different from the conventional machine learning approaches for wireless localization, in which the above differential RSS measurements are trivially used as the only input features, we introduce the parameterized geometrical representation for an affected link, which consists of its geometrical intercepts and differential RSS measurement. Parameterized geometrical feature extraction (PGFE) is performed for the affected links and the features are used as the inputs of ELM. The proposed PGFE-ELM for DFL is trained in the offline phase and performed for real-time localization in the online phase, where the estimated location of the target is obtained through the created ELM. PGFE-ELM has the advantages that the affected links used by ELM in the online phase can be different from those used for training in the offline phase, and can be more robust to deal with the uncertain combination of the detectable wireless links. Experimental results show that the proposed PGFE-ELM can improve the localization accuracy and learning speed significantly compared with a number of the existing machine learning and DFL approaches, including the weighted K-nearest neighbor (WKNN), support vector machine (SVM), back propagation neural network (BPNN), as well as the well-known radio tomographic imaging (RTI) DFL approach.

## 1. Introduction

Although some navigation systems, such as GPS and BeiDou, are popular for outdoor localization and navigation, they cannot function well in indoor environments, as signals from the satellites are often blocked by tall buildings [1]. Current indoor localization systems usually require that the entity to be localized be equipped with some kind of electronic device [2,3,4]. Device-free localization (DFL) was recently introduced as a new radio-frequency (RF)-based localization approach, where the target does not need to have any attached electronic device [5].

DFL can be applied to many applications, such as human health and medical care with the help of location detection and behavior analysis. As an example, the ability to monitor elderly people’s or rehabilitation activities and provide timely assistance depends highly on their location and activity information. When some abnormalities are identified (e.g., suddenly falling down and staying in the bed for too long), alarms should be triggered to inform the caregivers together with their location information. Such localization functions can be implemented by the conventional technologies, such as WiFi, RFID, or some other wearable devices, but these represent heavy burdens for the people involved, and cause privacy and comfort problems. Location and physiological index information are also important for the dementia patients who may get lost easily.

In DFL, radio transmitters (RTs) and radio receivers (RXs) are used as the sensors to sense the target collaboratively. As the original signal field will be changed when the target enters the monitoring area, the location of the target can be estimated by analyzing the changes of the signals, i.e., the differential received signal strength (RSS) measurements of the corresponding links in the network [6].

Although DFL can be applied in indoor and outdoor environments, it still suffers from non-line-of-sight, multi-path and fading phenomena, due to the uncertain and dynamic wireless propagation environment [7,8,9,10], therefore DFL remains a challenging research problem. Most existing approaches for DFL have their own merits and limitations. For example, the popular radio tomographic imaging (RTI) approach can provide relatively satisfactory performance, but it does not consider the negative impacts of the links that do not detect the target, and the weightings of voxels along one link are the same, which is not consistent with the actual situation [8,11].

It should be noted that although machine learning approaches have been used in localization field and can achieve satisfactory performance [12,13], they are rarely involved in DFL. Thus, it is worthwhile to study how to apply machine learning approaches to DFL and examine their performance.

Existing machine learning approaches, such as the back propagation neural network (BPNN) and support vector machine (SVM) [14], can easily get trapped in a local minimum and are time-consuming in the training phase. Thus, in this paper, we will introduce a novel machine learning approach, called extreme learning machine (ELM) [15], for DFL. ELM is an extension of the traditional training approaches of single-hidden layer feedforward neural networks (SLFNs), in which the hidden layer parameters are generated randomly in advance and only the output weights are required to be calculated optimally through the least-square approach, so it can achieve better generalization performance with higher accuracy, and is much faster than existing machine learning approaches [16,17,18]. In addition, it can provide a unified learning platform with widespread types of feature mapping problems and can be applied directly in regression, binary classification, and multiclass classification. Due to the aforementioned advantages, ELM has received much attention and has been used in many fields, such as face recognition [19,20], industrial production [21,22], human physical activity recognition [23,24], landmark recognition [25,26], and leukocyte image segmentation [27], etc.

Trivially, the differential RSS measurements can be used as the only inputs of the machine learning approaches. In order to obtain more accurate model for DFL, we introduce the parameterized geometrical representation for an affected link that can detect the target, which consists of its geometrical intercepts and differential RSS measurement, by treating the monitoring area as a 2-dimentioanl plane. Parameterized geometrical feature extraction (PGFE) is performed for the affected links and the features are used as the inputs of ELM.

The proposed PGFE-ELM for DFL is performed in two phases, i.e., the offline training phase and the online localization phase. During the offline training phase, the ELM is trained using the input features extracted through PGFE. During the online localization phase, the real-time location of the target will be estimated. In this paper, we will focus on the DFL problem for single target. The main contributions of this paper include: (1) ELM is introduced to DFL to improve the localization accuracy and computation speed; (2) the parameterized geometrical representation is defined for the affected link and the corresponding PGFE scheme is introduced to the proposed ELM; (3) the proposed PGFE-ELM approach is in nature robust to the uncertain combination of the detectable wireless links, which happens frequently in the real situations due to the uncertainties of the wireless propagation environment. 

The paper is organized as follows: the related work is introduced in Section 2. A brief introduction to ELM is given in Section 3. The proposed approach is detailed in Section 4, including PGFE and the PGFE-ELM-based DFL. Experimental results are reported in Section 5 to show the advantages of PGFE-ELM. Finally, conclusions and future work are given in Section 6.

## 2. Related Work

In the past decade, different approaches have been proposed for DFL, mainly including the RTI approach, the compressive sensing (CS) approach, the fingerprinting approach, and the geometric approach, etc.

RTI was proposed by Wilson et al. [8] for DFL by imaging the attenuation of the target. In RTI, the monitoring area was divided into voxels, and each voxel has different contributions for the signal attenuation of each link. Thus, the DFL is formulated as the linear ill-posed inverse problem, and solved by the regularization approach, such as the Tikhonov regularization [8,9]. Patwari et al. [28] applied measurement-based models to analyze and verify both the advantages and drawbacks of the correlated link shadowing in DFL. Wilson et al. [10] focused on the study of the RSS measurements in wireless networks to estimate the locations of both moving and stationary people, and showed the possibility of tracking more than one person. As the assumption in RTI that the weightings of the voxels along one link are the same is not practical in real situation, Lei et al. [11] proposed a geometry-based elliptical model with variable voxel weights and adopted an orthogonal matching pursuit algorithm to improve the localization accuracy. Banerjee et al. [29] used variance-based RTI (VRTI) in target tracking and localization, and showed that receiver attacks can be detected and the source of the unlawful activity can be identified with good precision. Kaltiokallio et al. [30] proposed an online recalibration approach through the finite-state machine, which allowed the system to adapt to the changes in the radio environment, and proposed a novel spatial weight model for RTI [31]. Zhao et al. [32] proposed least square variance-based radio tomography approach for DFL to reduce the impact of the environment noise. Although RTI can achieve relatively good performance, the determination of some parameters in regularization only depends on the experience, which is lack of the theoretical derivation and proof. In addition, it does not consider the negative impacts of the links that cannot detect the target, which may degrade the performance significantly.

As the weights of the voxels in RTI are space-domain sparse, the CS approach was applied to deal with the linear ill-posed inverse problem [33,34,35,36,37,38]. The time-of-flight measurements of the shadowed links were considered as the observation information, and a CS-based particle filter was proposed by making use of the space-domain scarcity and the time-domain scarcity [35]. The multiple frequencies and the multiple transmission power levels were used to enrich the link measurement information, and the location information of the target was reconstructed by a recursive CS approach [38].

The fingerprinting approach for DFL involves two phases, i.e., the offline phase and the online phase [39,40,41]. An accurate radio map is the key for achieving good localization accuracy, which is created in the offline phase by recording the differential RSS measurements of the links when the target locates at the reference points (RP) with known positions. In the online phase, the location of the target can be estimated by matching the created radio map. Aly et al. [42] leveraged an automation tool for fingerprint construction to study modified scenarios for WiFi-based DFL. Usually, the performance of fingerprinting approach may be tolerably acceptable, but the tedious calibration is a necessary step. So, it faces the challenges on how to building the accurate radio map efficiently.

The geometric approach estimates the location of the target through the geometric relationship between the target location and the links, therefore it is a model-based approach and does not need tedious offline calibration which is a must in the fingerprinting approach. From the perspective of the geometric approach, Zhang et al. [43,44] proposed a signal dynamic model to determine the properties of the RSS change behavior, together with three tracking approaches by the midpoint, the intersection, and the best-cover geometric calculation, respectively. The best-cover approach can achieve relatively higher localization accuracy than other two approaches, but tedious calibration is still required. Talapmpas et al. [45] proposed the multichannel geometric filter for DFL by using channel diversity to diminish the effects of multipath fading of the links, and improve the localization accuracy for DFL in cluttered environments.

A Bayesian approach was introduced for DFL by using the probabilistic observation information of the shadowed links, the constraint information of the non-shadowed links, and the prior probabilistic estimation information, to strengthen the robustness of the model and avoid the overfitting problem [46]. Savazzi et al. [47] studied the diffraction principle to deal with the average path loss and the fluctuations of the RSS measurements induced by the moving target, and proposed a modified stochastic Bayesian approach for real-time target localization.

As a machine learning approach, Chiang et al. [14] proposed a modified fuzzy SVM and applied it to DFL. The undergoing SVM involves a quadratic programming problem, which is computationally expensive. Thus, SVM-based DFL is time-consuming, and difficult to deal with the large number of data in DFL. Dong et al. proposed a joint learning approach for intrusion detection. However, it needs to identify problematic wireless devices that report wrong signal readings [48] and the computational burden is high. Although both the fingerprinting and machine learning approaches proposed for DFL are based on pattern matching, the machine learning approach needs to determine the relevant parameters to build the model for DFL that can be used in the online phase, while the fingerprinting approach is implemented by matching with the radio map established in the offline phase.

## 3. Extreme Learning Machine

**Figure 1:**
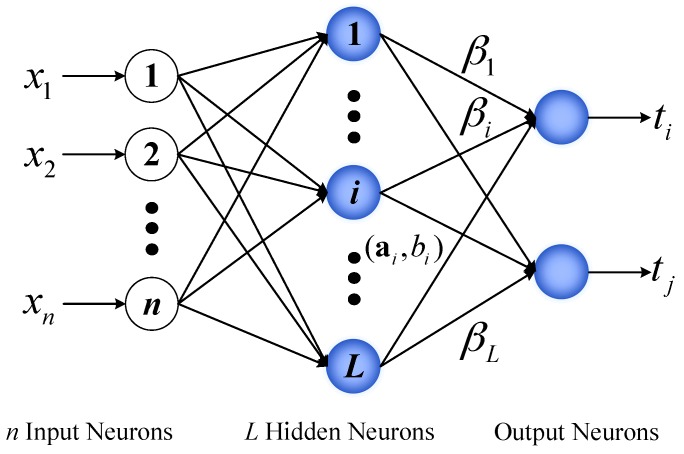
Basic structure of ELM.

In this section, the ELM theory is briefly introduced to facilitate the understanding of the proposed approach for DFL. ELM, which adopts a three-layer structure including the input layer, the hidden layer, and the output layer (see Figure 1), was originally proposed for SLFNs and was extended to the generalized SLFNs where the hidden layer need not be neuron-like [49]. Different from other traditional machine learning approaches, in ELM, the hidden layer parameters are pre-assigned randomly, the output weights can be analytically calculated through the least-square approach. It is proven that theoretically ELM has the universal approximation capability with any non-constant piecewise continuous activation function [50].

Suppose the number of hidden nodes is *L* in the hidden layer, the output of the hidden node *i* is described as $g(x;a_{i},b_{i})$ with $a_{i}$ and $b_{i}$ being the corresponding hidden parameters of this node, respectively, *i* = 1, …, *L*. For a given dataset ${\left\{(x_{i},t_{i})\right\}}_{i=1}^{N}\subset R^{n}\times R^{m}$, where $x_{i}$ is a *n*-dimensional input vector and $t_{i}$ is the corresponding *m*-dimensional observation vector. Its mapped feature vector is:

(1) $$h(x)=[g(x;a_{1},b_{1}),g(x;a_{2},b_{2}),...,g(x;a_{L},b_{L})],$$

The output function of ELM for generalized SLFNs with *L* hidden nodes can be represented by:

(2) $$f_{L}(x)=\sum_{i=1}^{L}\beta_{i}h_{i}(x)=h(x)\beta ,$$

where $\beta ={\left[\beta_{1},\beta_{2},...,\beta_{L}\right]}^{T}$ is the vector of the output weights connecting the hidden layer and the output.

Different from the traditional machine learning approaches, ELM aims to reach the smallest training error and the smallest norm of the output weights:

(3) $$Minimize:{\left\|\beta \right\|}_{p}^{\sigma_{1}}+C{\left\|H\beta -T\right\|}_{q}^{\sigma_{2}},$$

where $\sigma_{1}>0$, $\sigma_{2}>0$, p,q=0,(1/2),1,2,...,+∞, *C* is a user specified parameter and provides a tradeoff between the empirical risk minimization and the structural risk minimization, ***H*** is the hidden layer output matrix (randomized matrix):

(4) $$H=\left[\begin{array}{c} h(x_{1})\\ \vdots \\ h(x_{N}) \end{array}\right]=\left[\begin{array}{ccc} h_{1}(x_{1}) & \cdots & h_{L}(x_{1})\\ \vdots & \ddots & \vdots \\ h_{1}(x_{N}) & \cdots & h_{L}(x_{N}) \end{array}\right],$$

***T*** is the training data target matrix:

(5) $$T=\left[\begin{array}{c} t_{1}^{T}\\ \vdots \\ t_{N}^{T} \end{array}\right]=\left[\begin{array}{ccc} t_{11} & \cdots & t_{1m}\\ \vdots & \ddots & \vdots \\ t_{N1} & \cdots & t_{Nm} \end{array}\right].$$

The three-step learning process of ELM can be summarized as follows:

(1) Randomly assign the hidden layer parameters, i.e., $a_{i}$ and $b_{i}$.
(2) Calculate the hidden layer output matrix ***H***.
(3) Obtain the output weight vector:
  (6) $$\beta =H^{†}T,$$
  where $H^{†}$ is the Moore-Penrose generalized inverse of the matrix ***H***.

Usually, the resultant solution is equivalent to the ELM optimization solution with $\sigma_{1}=\sigma_{2}=p=q=2$, which can be mathematically written as:

(7) $$Minimize:L_{P_{ELM}}=\frac{1}{2}{\left\|\beta \right\|}^{2}+\frac{1}{2}C\sum_{i=1}^{N}\xi_{i}^{2}\;s.t.,\;h(x_{i})\beta =t_{i}-\xi_{i},i=1,...,N,$$

where $\xi_{i}=[\xi_{1,m},...,\xi_{i,m}]$ is the training error vector of the m output nodes with respect to the training sample $x_{i}$. Thus, the optimization problem can derive a stable solution with better generalization performance.

Then, based on the Karush-Kuhn-Tucker (KKT) theorem, we have:

(8) $$\beta =\{\begin{array}{cc} H^{T}{\left(\frac{I}{C}+H^{T}H\right)}^{-1}T, & N<L\\ {\left(\frac{I}{C}+H^{T}H\right)}^{-1}H^{T}T, & N>L \end{array},$$

where I is the unit matrix.

## 4. Proposed Approach

In this section, we will introduce the details of the proposed parameterized geometrical feature extraction based ELM (PGFE-ELM) for DFL.

### 4.1. Geometrical Represention of the Affected Link

We will firstly introduce the meaning of the affected link. Let ${\mathrm{RSS}}_{f}^{0}$ denote the RSS measurement of the link *f* when there is no any entity in the monitoring area, and ${\mathrm{RSS}}_{f}$ denotes the RSS measurement of the link *f* when the target enters the monitoring area. The differential RSS measurement of the link *f* can be expressed as:

(9) $$\Delta {\mathrm{RSS}}_{f}={\mathrm{RSS}}_{f}-{\mathrm{RSS}}_{f}^{0}.$$

**Figure 2:**
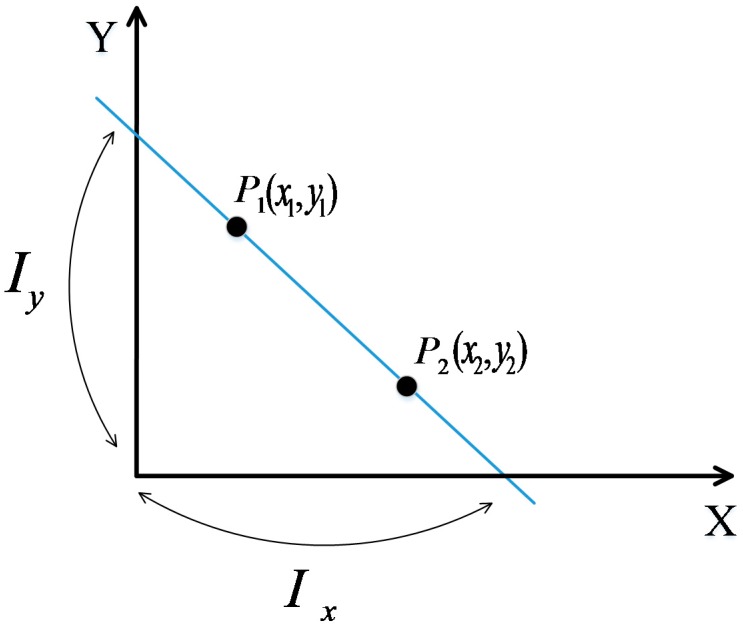
Intercepts of the straight line passing P_1_ and P_2_.

We call the links with distinct differential RSS measurements due to the presence of the target as the affected links. If we build a coordinate system for the monitoring area of the DFL, all the fixed nodes have their own corresponding coordinates. As shown in Figure 2, when a straight line passing through two points *P*_1_(*x*_1_, *y*_1_) and *P*_2_(*x*_2_, *y*_2_), and assuming $x_{1}\neq x_{2}$ and $y_{1}\neq y_{2}$, the x-axis intercept $I_{x}$ and the y-axis intercept $I_{y}$ of the straight line can be calculated by:

(10) $$I_{x}=\frac{x_{2}y_{1}-y_{2}x_{1}}{y_{1}-y_{2}},$$

(11) $$I_{y}=\frac{y_{2}x_{1}-x_{2}y_{1}}{x_{1}-x_{2}}.$$

**Figure 3:**
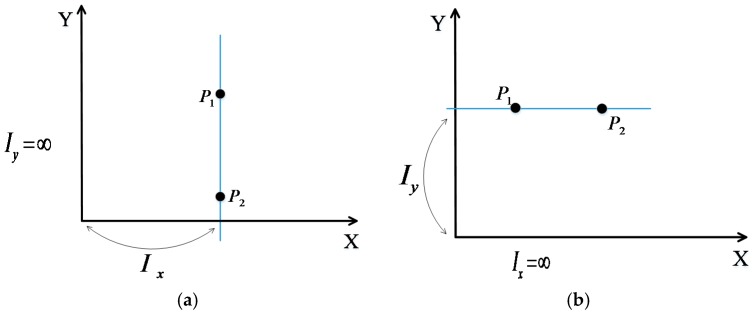
Particular situations of intercepts of the straight line parallels to the y-axis (**a**) the x-axis (**b**).

Particularly, when the straight line parallels to the y-axis or the x-axis (see Figure 3a,b), we can find that $I_{y}=\infty$ and $I_{x}=\infty$, respectively.

In the monitoring area, each affected link from one node to another node can be expressed using a straight line described by its geometrical representation, which consists of its x-axis intercept and y-axis intercept, as well as its differential RSS measurement.

### 4.2. PGFE-ELM Based DFL

**Figure 4:**
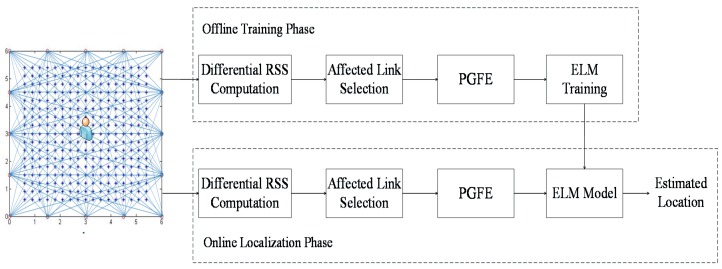
Framework of the PGFE-ELM based DFL.

The framework of the PGFE-ELM based DFL is shown in Figure 4. It involves two phases, the offline training phase and the online localization phase. During the offline training phase, reference points (RPs) are used for data collection and ELM training as the samples. Each RP is associated with a number of affected links, and the corresponding affected links of all the RPs can be used for providing the ELM training dataset.

For each RP, we firstly perform differential RSS computation. In order to reduce the computational overhead of ELM and avoid the overfitting problem, the affected link selection is necessary, where a number of affected links with relatively larger differential RSS measurements are selected. 

Feature extraction is very important to ELM, which can affect the performance of ELM significantly. Traditionally, the differential RSS measurements of the affected links are the only input features, which is not enough to create an accurate model. Thus, we will perform the parameterized geometrical representations of the selected affected links and the corresponding feature extraction using the proposed PGFE.

For the selected affected links, we can extract their x-axis intercepts and y-axis intercepts, together with their corresponding differential RSS measurements as the input features of ELM. Assume n affected links are selected, and then the input features for ELM can be represented as:

(12) $$\left\{I_{x1},I_{y1},\Delta {\mathrm{RSS}}_{1},I_{x2},I_{y2},\Delta {\mathrm{RSS}}_{2},...,I_{xn},I_{yn},\Delta {\mathrm{RSS}}_{n}\right\}.$$

**Figure 5:**
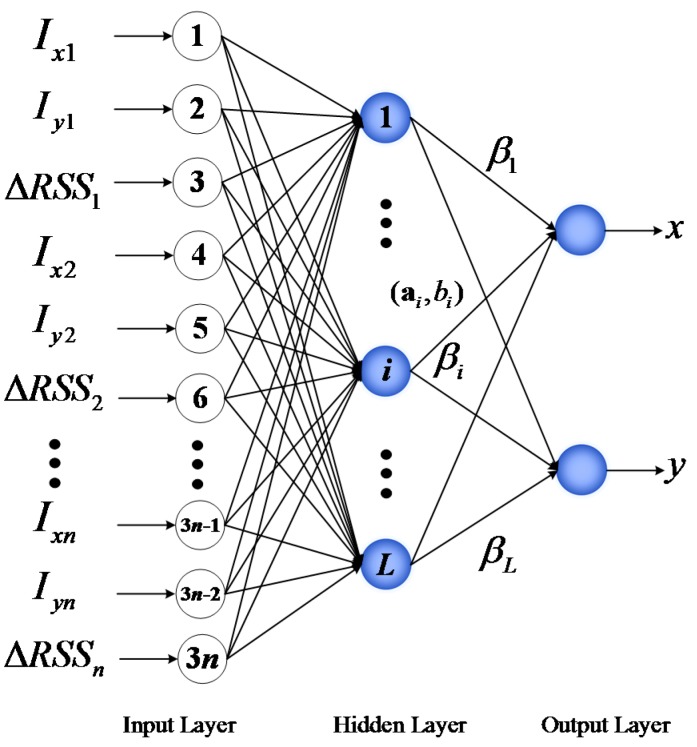
Schematic Diagram of PGFE-ELM.

As shown in Figure 5, in PGFE-ELM, the input features of ELM are extended from the only ΔRSS values of the affected links to the x-axis intercepts, y-axis intercepts and ΔRSS values of the affected links, and the outputs are the location estimation of the target. So the more accurate ELM can be trained with extended input features.

Assuming that there are *l* RPs, and each RP corresponds to n selected affected links, so the input matrix of ELM can be written as:

(13) $$Input_{ELM}={\left[\begin{array}{ccccccccc} I_{x1}^{1} & I_{y1}^{1} & \Delta {\mathrm{RSS}}_{1}^{1} & I_{x1}^{2} & I_{x1}^{2} & \Delta {\mathrm{RSS}}_{1}^{2} & \cdots & I_{x1}^{n} & \begin{array}{cc} I_{y1}^{n} & \Delta {\mathrm{RSS}}_{1}^{n} \end{array}\\ I_{x2}^{1} & I_{y2}^{1} & \Delta {\mathrm{RSS}}_{2}^{1} & I_{x2}^{2} & I_{x2}^{2} & \Delta {\mathrm{RSS}}_{2}^{2} & \cdots & I_{x2}^{n} & \begin{array}{cc} I_{y2}^{n} & \Delta {\mathrm{RSS}}_{2}^{n} \end{array}\\ \vdots & \vdots & \vdots & \vdots & \vdots & \vdots & \ddots & \vdots & \begin{array}{c} \begin{array}{cc} \begin{array}{c} \vdots \end{array} & \vdots \end{array} \end{array}\\ I_{xl}^{1} & I_{yl}^{1} & \Delta {\mathrm{RSS}}_{n}^{1} & I_{xl}^{2} & I_{xl}^{2} & \Delta {\mathrm{RSS}}_{l}^{2} & \cdots & I_{xl}^{n} & \begin{array}{cc} I_{yl}^{n} & \Delta {\mathrm{RSS}}_{l}^{n} \end{array} \end{array}\right]}_{l\times 3n},$$

and the input weights with *L* hidden nodes are:

(14) $$\omega =\left[\begin{array}{c} \omega_{1}\\ \omega_{2}\\ \vdots \\ \omega_{n} \end{array}\right]={\left[\begin{array}{cccc} \omega_{1}^{1} & \omega_{1}^{2} & \cdots & \omega_{1}^{3n}\\ \omega_{2}^{1} & \omega_{2}^{2} & \cdots & \omega_{2}^{3n}\\ \vdots & \vdots & \ddots & \vdots \\ \omega_{n}^{1} & \omega_{n}^{2} & \cdots & \omega_{n}^{3n} \end{array}\right]}_{L\times 3n}.$$

The output can be expressed as:

(15) $$Output_{ELM}=T={\left[\begin{array}{ll} x_{1} & y_{1}\\ x_{2} & y_{2}\\ \vdots & \vdots \\ x_{l} & y_{l} \end{array}\right]}_{l\times 2}.$$

According to (6), the output weight of ELM will be:

(16) $$\beta =H^{†}T={\left[\begin{array}{ll} \beta_{1}^{1} & \beta_{1}^{2}\\ \beta_{2}^{1} & \beta_{2}^{2}\\ \vdots & \vdots \\ \beta_{L}^{1} & \beta_{L}^{2} \end{array}\right]}_{L\times 2}.$$

During the online localization phase, the differential RSS computation and affected link selection are performed to select the appropriate number of the affected links for the target. After that, all the selected affected links are represented geometrically, and the x-axis intercepts, y-axis intercepts and the corresponding differential RSS measurements constitute the testing dataset together. Finally, the trained ELM model outputs the location of the target given the extracted features of the affected links.

PGFE-ELM has the following two advantages: (1) during the offline training phase, for providing training samples, the combination of the *n* affected links associated each RP can be different from sample to sample; (2) the affected links used by ELM in the online localization phase can be different from those used for training in the offline training phase. As a result, PGFE-ELM is more robust to deal with the uncertain combination of the detectable wireless links, both during the offline training phase and the online localization phase.

## 5. Performance Verification

### 5.1. Experiments Settings

We build the wireless network using CC2530 ZigBee nodes, which are based on the IEEE 802.15.4 standard and operate in the 2.4 GHz frequency band. RPs are set up uniformly in the monitoring area. Two different experiments are performed to evaluate the proposed approach, respectively for the outdoor environment and the indoor environment. 

In the experiments, we choose the weighted *K*-nearest neighbor (WKNN), SVM, and BPNN for comparison to verify the performance of the proposed PGFE-ELM. As mentioned in Section 4.1, we set $I_{y}=9999$ and $I_{x}=9999$ in the following experiments for the two situations in Figure 3a,b. After data collection, the localization algorithms are carried out in Matlab 2012a environment running in an Inter i5 3.2 GHz CPU and 4G RAM. In addition, we use the following localization accuracy (17) as the evaluation criteria:

(17) $$Accuracy=\frac{1}{z}\sum_{i=1}^{z}\sqrt{{\left(x_{i}-x_{i0}\right)}^{2}+{\left(y_{i}-y_{i0}\right)}^{2}},$$

where $\left(x_{i},y_{i}\right)$ is the predicted coordinates, and $\left(x_{i0},y_{i0}\right)$ is the real coordinates of the *i*th testing point (TP), and *z* is the number of the TPs.

In the performance evaluation processes, the data from RPs is used for training and the data from TPs is used for testing. Each RP provides one training sample, and each TP provides one testing sample. It should be noted that we use the same person to collect the training data and the testing data. The RSS are sampled at the 5-min interval in the working days, and the RSS values of all the RPs and TPs are collected 100 times, and the average values of these RPs and TPs consist of the training dataset and the testing dataset, respectively.

### 5.2. Experimental Performance Evaluation for Outdoor Environment

**Figure 6:**
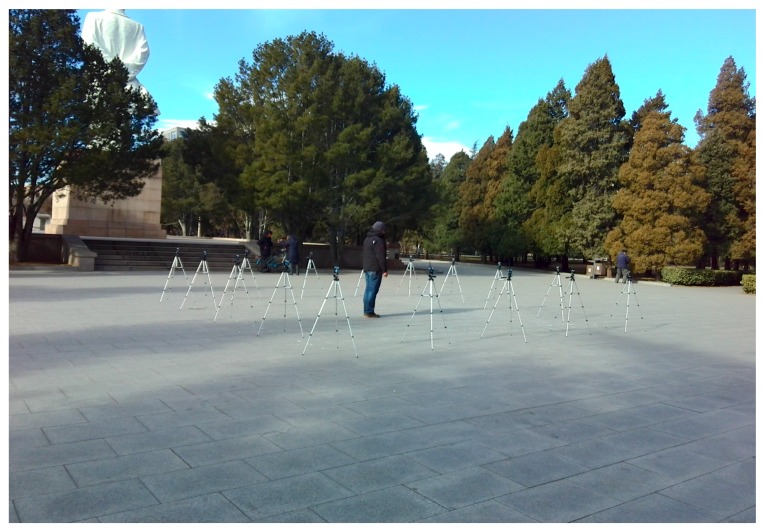
Data collecting process for the outdoor environment.

**Figure 7:**
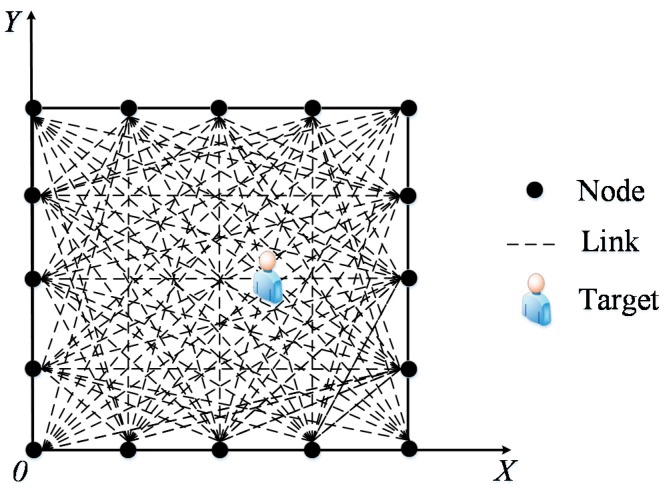
Coordinate system of the monitoring area**.**

The outdoor environment experiment was set up on the campus of the University of Science and Technology Beijing. As shown in Figure 6, the monitoring area is a 6 m × 6 m square, with 16 nodes placed along its boundary and the adjacent node distances of 1.5 m. In the experiments, for two RP distribution scenarios are examined, one with 0.3 m space between adjacent RPs (i.e., 0.3 m RP spaced scenario) and another one with 0.6 m space between adjacent RPs (i.e., 0.6 m RP spaced scenario). In the first scenario, there are 289 RPs in total, whereas in the second scenario there are 81 RPs in total. In order to implement the proposed PGFE-ELM, we build a coordinate system for the monitoring area, in which the x-axis and the y-axis overlap the two edges of the monitoring area (see Figure 7).

**Figure 8:**
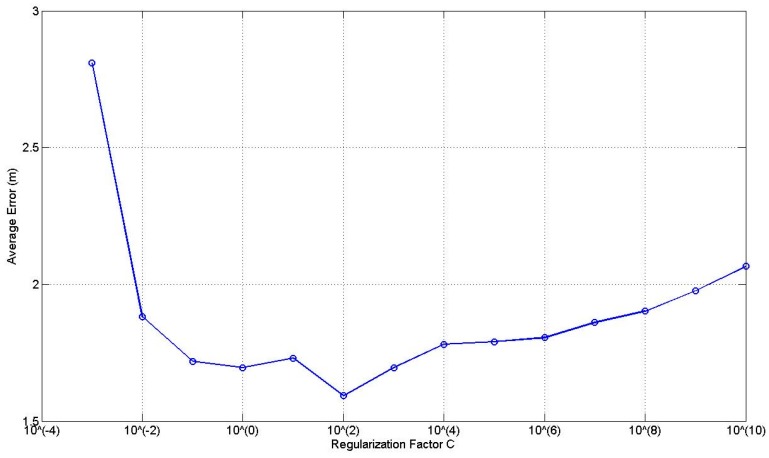
Effect of the regularization factor *C*.

There are two tuning parameters in ELM (sigmoid function is used as the activation function), i.e., the regularization factor *C* and the number of hidden nodes *L*. Figure 8 illustrates the average error curve using all the links at the 0.3 m RP spaced scenario with respect to *C* given *L* = 10. It can be found that the average error curve presents a parabolic shape with the increase of *C* and obtains the smallest average error when *C* = 10^2^. Therefore, we choose *C* = 10^2^ in the following experiments.

**Figure 9:**
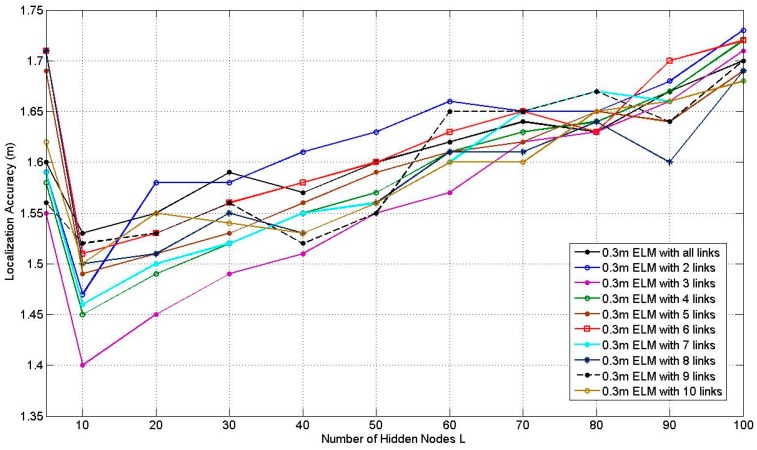
Results of PGFE-ELM at 0.3 m RP spaced scenario.

Figure 9 illustrates how the average localization accuracy of PGFE-ELM changes with the increase of the number of hidden nodes from 5 to 100, for the 0.3 m RP spaced scenario with the number of the affected links from 2 to 10 and all the affected links. According to Figure 9, it can be found that the best average localization accuracies can be achieved when *L* = 10, no matter how many affected links are selected. The situation when *L* = 10 with 3 selected affected links can obtain the best average localization accuracy, which equals to 1.40 m.

**Figure 10:**
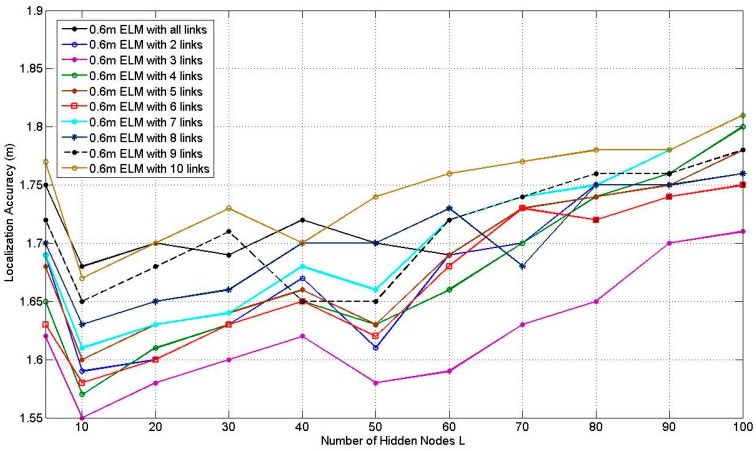
Results of PGFE-ELM at 0.6 m RP spaced scenario.

Figure 10 illustrates the average localization accuracy of PGFE-ELM when the RP is 0.6 m spaced scenario. Similar to Figure 9, the corresponding best average localization accuracies are obtained when *L* = 10. The situation when *L* = 10 with 3 affected links can obtain the best average localization accuracy, which equals to 1.55 m. Comparing Figure 9 and Figure 10, we can find that the average localization accuracies of most situations in Figure 9 with the 0.3 m RP spaced scenario are better than the corresponding situations in Figure 10. Whereas the average localization accuracies of in Figure 10 with the 0.6 m RP spaced scenario is worse but with less calibration overhead. 

Normally the affected link is identified by comparing the differential RSS measurement with a predefined threshold. However, due to the uncertainties of the experimental environments, such threshold may introduce false detection. Differently, PGFE-ELM compares the differential RSS measurements of all the links and selects the given number of affected links from the most significant ones. As it is difficult to know the optimal number of the affected links in PGFE-ELM, we examine this by experiments.

**Figure 11:**
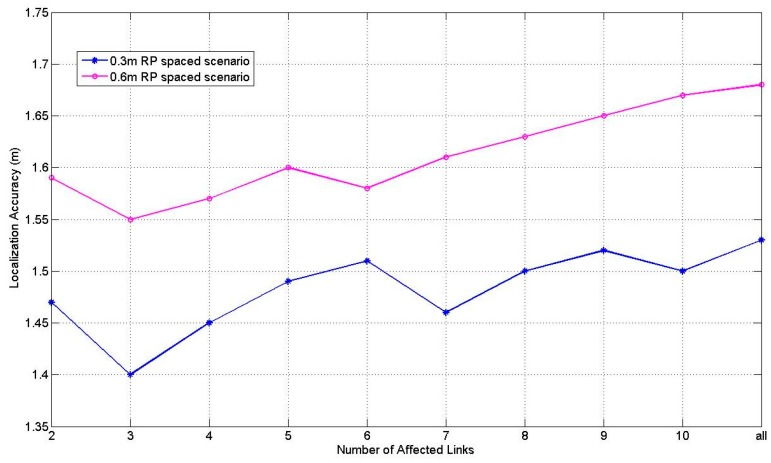
Performance of PGFE-ELM against different number of affected links with the number of hidden nodes 10.

Figure 11 illustrates the comparison results of PGFE-ELM accuracy versus the number of affected links with the number of hidden nodes 10 which is showed best performance in Figure 9 and Figure 10. We can find that PGFE-ELM achieves the best localization accuracy in both of the two RP spaced scenarios when the number of affected links is set as 3. The main reasons are that: if the assigned number of the affected links is set as 2, the number of links may be not enough for estimating the target location; whereas if the assigned number of the affected links is too large, some links may be identified as affected links incorrectly and subsequently degrade the localization performance.

**Figure 12:**
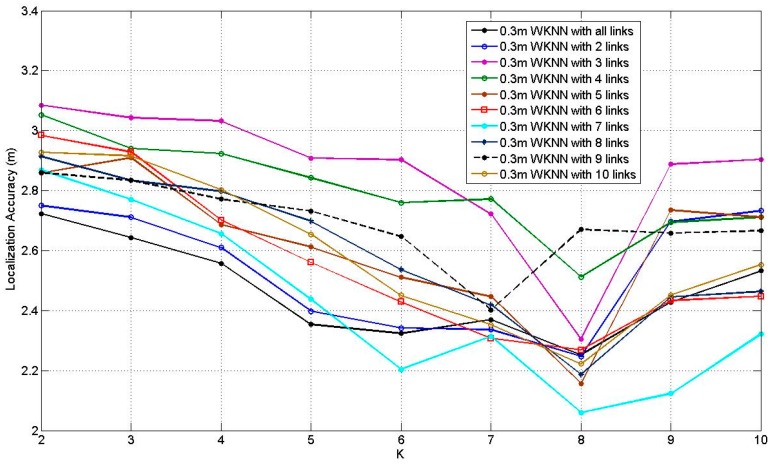
Results of WKNN at 0.3 m RP spaced scenario.

**Figure 13:**
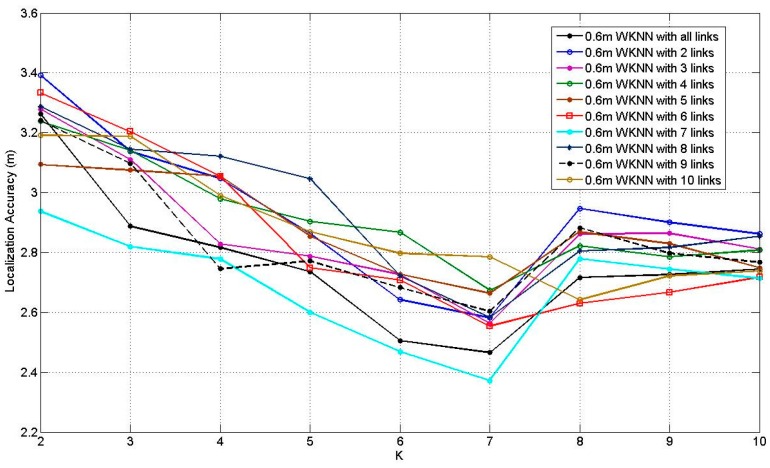
Results of WKNN at 0.6 m RP spaced scenario.

There is only one tuning parameter in WKNN, i.e., the number of neighbors *K*. Figure 12 illustrates the how the average localization accuracy of WKNN at 0.3 m RP spaced scenario changes when *K* increases from 2 to 10, with the number of the affected links from 2 to 10 and all the affected links. According to Figure 12, it can be found that all the curves present the downtrends before *K* = 8 and most of them rise after *K* = 8. The situation of *K* = 8 with 7 affected links obtains the best average localization accuracy, which equals to 2.06 m. Figure 13 illustrates the results of WKNN at 0.6 m RP spaced scenario, when increasing *K* from 2 to 10. Similar to Figure 12, all the curves present the downtrends before *K* = 7 and most of them rise after *K* = 7. The situation of *K* = 8 with 7 affected links obtains the smallest error, which equals to 2.37 m. Similar to ELM, in the 0.3 m RP spaced scenario, WKNN can achieve better average localization accuracy compared with the 0.3 m RP spaced scenario.

**Figure 14:**
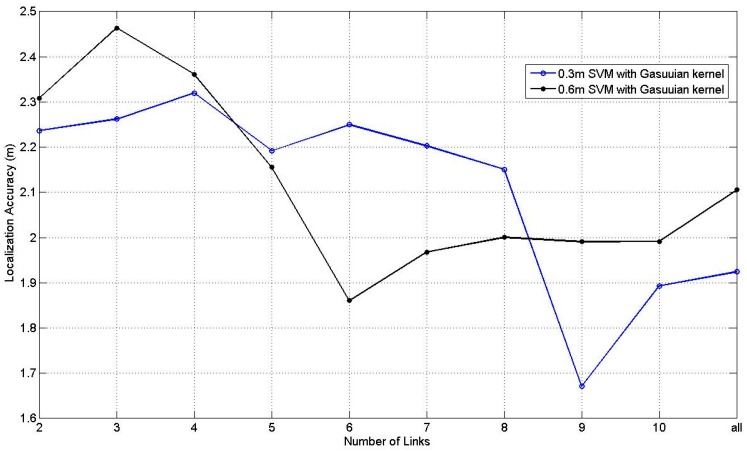
Results of SVM at 0.3 m and 0.6 m RP spaced scenarios.

Figure 14 illustrates the results of SVM with Gaussian kernel at 0.3 m and 0.6 m RP spaced scenarios s with the number of the affected links changing from 2 to 10 and all the affected links. All the parameters in SVM are obtained through the cross validation. According to Figure 14, it can be found that the situation of 0.6 RP spaced scenario is relatively smoother than the situation of 0.3 m RP spaced scenario with tiny fluctuation, and situation with 9 affected links at 0.3 m RP spaced scenario obtains the best average localization accuracy, which equals to 1.67 m.

**Figure 15:**
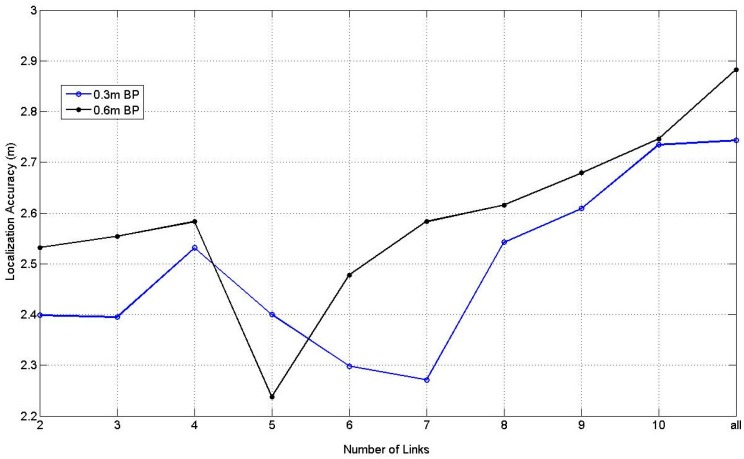
Results of BPNN at 0.3 m and 0.6 m RP spaced scenarios.

Figure 15 illustrates the results of BPNN with one hidden layer and 10 hidden nodes at 0.3 m and 0.6 m RP spaced scenarios with the changing of the number of the affected links from 2 to 10 and all the affected links. In Figure 15, the situation with 5 affected links at 0.6 m spaced scenario obtains the best average localization accuracy, which equals to 2.23 m.

Table 1 and Table 2 demonstrate the time consumption of ELM, WKNN, SVM and BPNN with 3, 5, 7, 9 and all links at 0.3 m RP spaced scenario and 0.6 m RP spaced scenario, respectively. We can find that obviously ELM is much faster than other machine learning approaches, especially in training time. For example, ELM is thousands of times faster than SVM and hundreds of times faster than BPNN at 0.3 m RP spaced scenario. Furthermore, the testing time of ELM equals to or is close to 0 s in most situations. Evidently, in terms of time consumption, with the increase of data quantity, the advantage of ELM will be more significant.

Furthermore, the best and worst localization accuracy among TPs in different situations is listed in Table A1, Table A2, Table A3, Table A4 and Table A5 in the Appendix A, respectively. For ELM and BPNN, the number of hidden nodes *L* is set as 10; for WKNN, *K* is set as 8 in the 0.3 m scenario and 7 in the 0.6 m scenario. We can find that in some situations, the best localization accuracy of ELM and SVM are even under 0.1 m. In addition, the worst localization accuracy in different situations of ELM is about 3 m, and other approaches can be high as 5 m. These results indicate that ELM is more robust than other machine learning approaches.

According to the above figures and tables, we can find that the results of the 0.3 m RP spaced scenario of all the four machine learning approaches are better than their corresponding results of the 0.6 m RP spaced scenario in most situations, which indicates that 0.3 m between adjacent RPs is more suitable than the case with 0.6 m. Furthermore, if we use all the affected links in the experiment, all the situations cannot obtain their own best average localization accuracies, which indicates that choosing the appropriate number of the affected links is important and can reduce the computation burden of the models and improve the localization accuracy efficiently. Finally, we also can find that all of the localization accuracy of PGFE-ELM in different situations is much better than WKNN, SVM, and BPNN, which shows its excellent generalization performance. It should note that the best average localization accuracy of these approaches is 1.40 m, which is achieved by PGFE-ELM with the 0.3 m RP spaced.

In order to verify the validity of the proposed PGFE, we perform the experiment using the original dataset only with the differential RSS values. Table 3 lists the comparison results of the four machine learning approaches with and without PGFE in the situation of 3 affected links of the 0.3 m RP spaced scenario. It can be found that the results with PGFE are much better than the corresponding ones without PGFE, WKNN and SVM can achieve about 1 m improvement when PGFE is used.

### 5.3. Experimental Performance Evaluation for Indoor Environment

**Figure 16:**
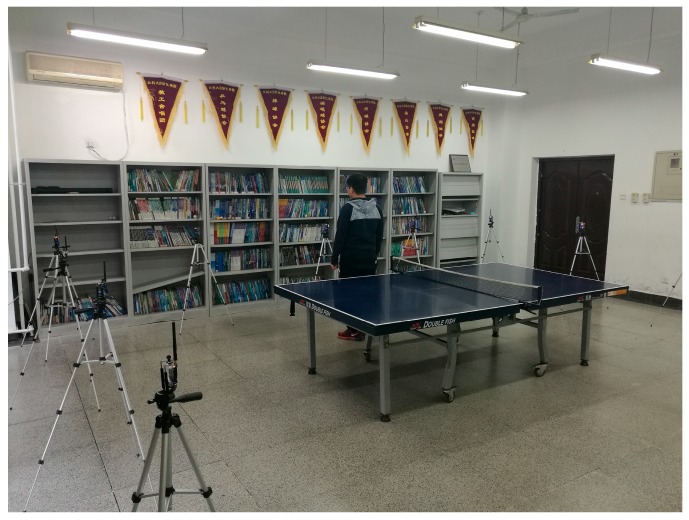
Indoor experiment setup for DFL algorithm comparison.

The indoor environment experiment was set up in a staff activity room. As shown in Figure 16, the monitoring area is a 6 m × 6 m square, with 16 nodes placed along its boundary and the adjacent node distances of 1.5 m. There is a table tennis table inside the area as an obstacle. Similar to the outdoor experiment, two RP distribution scenarios are examined, one with 0.3 m spaced between adjacent RPs (i.e., the 0.3 m RP spaced scenario) and another one with 0.6 m spaced between adjacent RPs (i.e., the 0.6 m RP spaced scenario).

**Figure 17:**
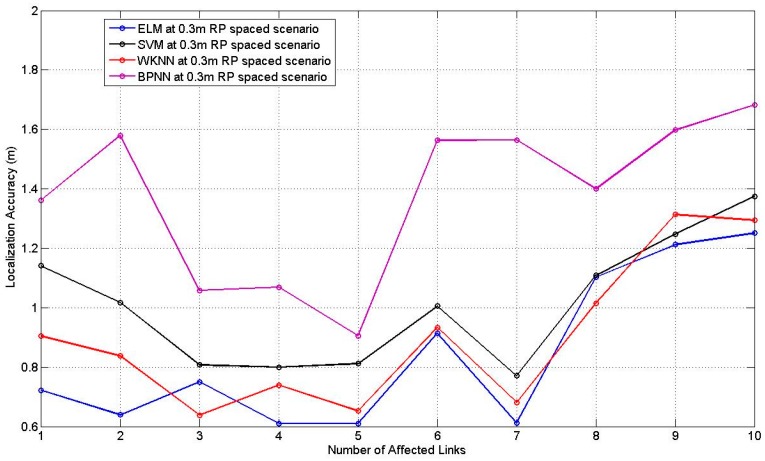
Comparison result with different numbers of affected links for 0.3 m RP spaced scenario.

**Figure 18:**
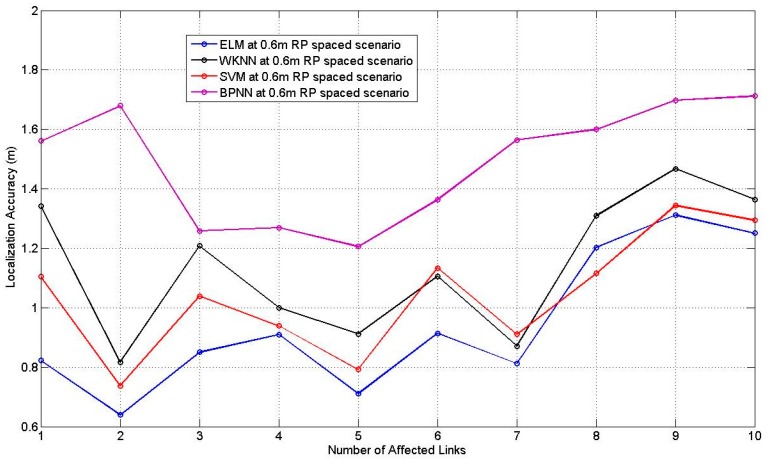
Comparison result with different numbers of affected links for 0.6 m RP spaced scenario.

The number of the hidden nodes of ELM and BPNN is set as 10, *K* of WKNN is set as 8, the corresponding parameters of SVM are determined through the cross validation. Figure 17 and Figure 18 illustrate the comparison results of the four machine learning approaches for the 0.3 m and the 0.6 m spaced scenarios, respectively, with the corresponding best and worst localization accuracy among TPs listed in Table A6, Table A7 and Table A8 in the Appendix A. 

Table 4 and Table 5 show the time consumption result of the approaches. We can find that the average localization accuracy of BPNN is the worst, ELM obtains the best performance in most situations, and sometimes the performance of SVM is similar to ELM. In terms of time consumption, ELM has still shown outstanding advantage, and the testing time equals to 0 s in many situations (see Table 4 and Table 5). The testing time of SVM is roughly the same as ELM, but its training time is hundreds of times slower than ELM.

## 6. Discussion

Although the proposed PGFE-ELM is more robust than other machine learning-based DFL approaches, its localization accuracy depends on the original data quality. We can find that the average localization accuracy of the outdoor experiment is more than 1 m, but the best average localization accuracy of the indoor experiment is about 0.6 m. The main reason we think is that we collected the outdoor environment data in a winter, windy and cold weather, which may have great impacts on the data quality, as the weather can affect the device performance and data transmission characteristic seriously.

According to the above experiments, PGFE-ELM obtains the best performance in both outdoor and indoor environments, especially in terms of learning speed, which is hundreds and even thousands of times faster than others. Thus, compared with other machine learning approaches, PGFE-ELM is more suitable for DFL. In order to verify the proposed PGFE-ELM further, we performed the comparison between PGFE-ELM and RTI. The experimental data was obtained from the SPAN Lab of the University of Utah [51]. There are 35 location points in the dataset. We randomly choose 30 location points for training PGFE-ELM and five location points for testing. According to the comparison result, we can find that the average localization accuracy of PGFE-ELM and RTI are 0.7183 feet and 0.8244 feet, respectively, which indicate that PGFE-ELM can achieve better performance than RTI. 

Compared with PGFE-ELM, RTI is easy for use as it does not need the offline phase for data collection and training, but its parameters highly depend on the locations and the number of the nodes and must be reset when the locations and the number of the nodes are changed. However, when such changes happen, the trained PGFE-ELM can still be used directly without training again, so the practicability of PGFE-ELM is better than RTI.

## 7. Conclusions

In this paper, the parameterized geometrical representation of the affected link is introduced, and a novel ELM is proposed to implement the fast and accurate DFL based on the parameterized geometrical feature extraction from the affected links. The experimental results show that the proposed PGFE-ELM can achieve much better localization accuracy and faster learning speed than three existing machine learning approaches WKNN, SVM and BPNN, it can also achieve better localization accuracy than existing RTI approach. The proposed PGFE-ELM is developed based on the original ELM, further study is required to develop the on-line ELM approach for DFL to deal with the dynamic communication environment. Also this paper only considers localization problem for single target, future work can be done to address the multi-target localization problem.

## Figures and Tables

**Table 1:** Time consumption for 0.3 m RP spaced scenario.

Approach	Training Time (s)					Testing Time (s)				
	3	5	7	9	All	3	5	7	9	All
ELM	0.0128	0.0156	0.0161	0.0132	0.0172	0	0	0.0011	0.0015	0.0312
WKNN	-	-	-	-	-	0.4530	0.3588	0.3900	0.4056	0.3744
SVM	13.8869	14.7421	14.9557	15.0245	15.3069	0.0312	0.0311	0.0624	0.0613	0.0601
BPNN	2.6052	2.8236	2.9016	3.7752	3.7994	0.1245	0.0624	0.1560	0.0936	0.0983

**Table 2:** Time consumption for 0.6 m RP spaced scenario.

Approach	Training Time (s)					Testing Time (s)				
	3	5	7	9	All	3	5	7	9	All
ELM	0.0109	0.0103	0.0157	0.0155	0.0175	0	0	0	0.0019	0.0053
WKNN	-	-	-	-	-	0.3432	0.3744	0.3901	0.3276	0.3220
SVM	3.0420	2.9328	2.7456	2.6832	2.9172	0.0156	0.0319	0.0622	0.0411	0.0412
BPNN	0.8112	0.8736	0.9360	1.2792	1.8728	0.0378	0.0624	0.0468	0.0221	0.0936

**Table 3:** Comparison results of ELM, WKNN, SVM and BPNN with and without PGFE.

Approach	Accuracy (m)	Approach	Accuracy (m)
ELM (*L* = 10, without PGFE)	2.03	PGFE-ELM (*L* = 10)	1.40
WKNN (*K* = 10, without PGFE)	4.12	PGFE-WKNN (*K* = 10)	2.92
SVM (without PGFE)	2.91	PGFE-SVM	2.26
BPNN (*L* = 10, without PGFE)	3.12	PGFE-BPNN (*L* = 10)	2.39

**Table 4:** Time consumption for 0.3 m RP spaced scenario.

Approach	Training Time (s)					Testing Time (s)				
	3	5	7	9	All	3	5	7	9	All
ELM	0.0138	0.0159	0.0147	0.0212	0.0330	0	0	0.0051	0.0027	0.0511
WKNN	-	-	-	-	-	0.5693	0.5558	0.6780	0.6431	0.9745
SVM	11.6971	13.3375	13.7258	13.8909	15.3069	0.0312	0.0368	0.0553	0.0669	0.0690
BPNN	2.9098	2.8125	3.1052	3.6775	3.8989	0.1545	0.1629	0.0933	0.1296	0.1393

**Table 5:** Time consumption for 0.6 m RP spaced scenario.

Approach	Training Time (s)					Testing Time (s)				
	3	5	7	9	All	3	5	7	9	All
ELM	0.0089	0.0093	0.0119	0.0156	0.0175	0	0	0	0	0.0029
WKNN	-	-	-	-	-	0.3139	0.3566	0.3989	0.3370	0.3892
SVM	4.0285	3.3687	3.8091	2.1729	5.9095	0.0186	0.0412	0.0587	0.0419	0.0612
BPNN	0.9122	0.9663	0.9961	1.3722	1.7711	0.0351	0.0771	0.0375	0.0122	0.0731

## Appendix A

In this appendix, the localization accuracy among TPs of the four machine learning approaches at 0.3 m and 0.6 m spaced scenarios in outdoor and indoor environments are listed in Table A1, Table A2, Table A3, Table A4, Table A5, Table A6, Table A7 and Table A8.

**Table A1:** Best localization accuracy among TPs of ELM, WKNN, SVM and BPNN at 0.3 m spaced scenario of outdoor environment (m).

Approach	Number of Links									
	2	3	4	5	6	7	8	9	10	All
ELM	0.3406	0.2324	0.0615	0.2463	0.4031	0.2074	0.4060	0.0694	0.2941	0.5155
WKNN	0.8250	0.2361	0.5011	0.5230	0.4441	0.4998	0.2007	0.2752	0.5125	0.5731
SVM	0.2107	0.6580	0.3419	0.0975	0.0285	0.0228	0.0937	0.1999	0.1014	0.2828
BPNN	0.6642	0.4730	0.4369	0.8897	0.6539	0.3722	0.2866	0.7086	0.7951	0.4424

**Table A2:** Worst localization accuracy among TPs of ELM, WKNN, SVM and BPNN at 0.3 m spaced scenario of outdoor environment (m).

Approach	Number of Links									
	2	3	4	5	6	7	8	9	10	All
ELM	3.3620	3.2820	3.5700	3.4262	3.2371	2.9080	3.1547	3.2219	3.3510	3.0893
WKNN	3.6594	5.6385	5.2652	4.9193	4.9196	5.9020	5.8946	5.4341	5.8058	5.1679
SVM	5.9059	5.7051	5.7841	5.4635	4.8593	6.4115	5.3550	4.9691	5.2585	3.1113
BPNN	4.5499	4.2910	5.1657	5.2045	4.8855	5.0000	5.5551	4.9261	4.6214	5.6044

**Table A3:** Best localization accuracy among TPs of ELM, WKNN, SVM and BPNN at 0.6 m spaced scenario of outdoor environment (m).

Approach	Number of Links									
	2	3	4	5	6	7	8	9	10	All
ELM	0.5254	0.1505	0.5940	0.2037	0.2414	0.2753	0.2000	0.3125	0.3989	0.6993
WKNN	0.2070	0.2112	0.2567	0.2765	0.5116	0.2091	0.2207	0.0379	0.3967	0.6500
SVM	0.8823	0.0297	0.0850	0.3426	0.6738	0.0778	0.0991	0.1105	0.0350	0.1942
BPNN	0.6934	0.3937	0.9362	0.6055	0.2676	1.1450	0.5173	0.1836	0.6159	4.1873

**Table A4:** Worst localization accuracy among TPs of ELM, WKNN, SVM and BPNN at 0.6 m spaced scenario of outdoor environment (m).

Approach	Number of Links									
	2	3	4	5	6	7	8	9	10	All
ELM	4.7508	3.3681	4.9089	3.5675	3.4029	3.9213	3.2558	3.0006	3.6556	2.8699
WKNN	5.8599	5.5961	5.4407	4.8268	5.3352	5.4734	5.1885	5.4206	5.4736	4.7058
SVM	5.7051	5.8537	5.9274	5.3763	3.9187	4.3149	4.9691	3.2758	3.2575	3.2997
BPNN	5.8168	5.1911	5.9213	5.6006	4.5553	5.2982	4.4715	3.9201	5.9385	1.0004

**Table A5:** Best localization accuracy among TPs of ELM, WKNN, SVM and BPNN at 0.3 m spaced scenario of indoor environment (m).

Approach	Number of Links									
	2	3	4	5	6	7	8	9	10	All
ELM	0.2173	0.1055	0.1551	0.1831	0.2028	0.1633	0.1169	0.1267	0.1079	0.1680
WKNN	0.5659	0.1896	0.2558	0.2597	0.2630	0.6511	0.4542	0.5298	0.1290	0.1963
SVM	0.2002	0.1336	0.2626	0.4257	0.1336	0.4933	0.0998	0.7889	0.1639	0.2896
BPNN	0.1887	0.7983	0.7153	0.7008	0.6508	1.0237	0.7199	0.2528	0.6930	0.6865

**Table A6:** Worst localization accuracy among TPs of ELM, WKNN, SVM and BPNN at 0.3 m spaced scenario of indoor environment (m).

Approach	Number of Links									
	2	3	4	5	6	7	8	9	10	All
ELM	2.6123	1.8698	2.7878	3.2365	2.6145	2.6931	2.5100	3.1070	3.3961	2.7867
WKNN	3.2533	2.3332	2.5981	3.3536	2.7689	2.6109	2.8576	3.3098	3.3450	2.7911
SVM	3.0265	2.0986	2.7523	3.0158	2.6539	2.1983	3.3038	3.1260	3.2036	2.7009
BPNN	3.6698	2.6155	3.2256	3.1098	2.5502	3.2873	2.3008	3.0050	2.9032	2.9995

**Table A7:** Best localization accuracy among TPs of ELM, WKNN, SVM and BPNN at 0.6 m spaced scenario of indoor environment (m).

Approach	Number of Links									
	2	3	4	5	6	7	8	9	10	All
ELM	0.3110	0.2098	0.3055	0.3081	0.2059	0.2199	0.1208	0.1989	0.1233	0.2001
WKNN	0.6978	0.2323	0.3679	0.4251	0.3782	0.7598	0.6630	0.7895	0.1300	0.2981
SVM	0.3028	0.2365	0.3982	0.5530	0.1695	0.5333	0.1005	0.7190	0.1328	0.2550
BPNN	0.7835	0.6588	0.6960	0.6452	0.3640	0.9980	0.8899	0.3025	0.5899	0.6833

**Table A8:** Worst localization accuracy among TPs of ELM, WKNN, SVM and BPNN at 0.6 m spaced scenario of indoor environment (m).

Approach	Number of Links									
	2	3	4	5	6	7	8	9	10	All
ELM	2.8596	2.2980	2.8878	3.5223	2.5630	2.7815	2.7059	3.0009	3.5630	2.7811
WKNN	3.3536	2.5236	2.7971	3.6393	2.8896	2.9103	2.9982	3.5008	3.5673	2.9991
SVM	3.0095	2.3325	2.9633	3.2321	2.7520	2.5356	3.5029	3.3308	3.3569	3.0007
BPNN	3.8998	2.7892	3.6630	3.2079	2.8535	3.5505	2.4936	3.3291	2.7851	3.3332
